# Primary spindle cell sarcoma of the heart treated with carbon-ion radiotherapy: Case report

**DOI:** 10.1016/j.ijscr.2020.09.160

**Published:** 2020-09-25

**Authors:** Ikuko Shibasaki, Shigeru Toyoda, Yusuke Takei, Masayuki Chida, Hirotsugu Fukuda

**Affiliations:** a Department of Cardiac and Vascular Surgery, Dokkyo Medical University, Tochigi, Japan; b Department of Cardiovascular Medicine, Dokkyo Medical University, Tochigi, Japan; c Department of General Thoracic Surgery, Dokkyo Medical University, Tochigi, Japan

**Keywords:** Primary cardiac tumor, Spindle cell sarcoma, Carbon-ion radiotherapy

## Abstract

**Introduction:**

Primary cardiac sarcoma is a rare malignant cardiac neoplasm with a poor prognosis. No evidence-based guidelines exist regarding surgical treatment management, although some case reports have been published. We report the outcome of a patient with spindle cell sarcoma of the heart treated postoperatively with carbon-ion radiotherapy and chemotherapy.

**Presentation of case:**

A 16-year-old female patient was noted to have abnormal electrocardiogram results for 2 consecutive years at a high school medical check. She was referred to our hospital and a cardiac tumor in the left atrium was suspected. The preoperative diagnosis was myxoma, but abnormalities were also observed macroscopically during surgery, and a diagnosis of spindle cell sarcoma was made by rapid intraoperative pathological analysis. We resected the tumor as much as possible with surgery, but the tumor in the left lower pulmonary vein could not be respected. We performed carbon-ion radiotherapy postoperatively. She experienced recurrence 1 year later and tumor growth 2 years later. We considered additional surgical treatment, but eventually selected chemotherapy. She died 4 years and 7 months after initial surgery.

**Conclusion:**

Spindle cell sarcoma is a rare disease with a poor prognosis. Although no optimal treatment method has been established, in the present case, incomplete surgical resection and postoperative carbon-ion radiotherapy were performed, yielding a relatively long survival period.

## Highlights

• Primary cardiac tumors are rare. Seventy-five percent of the tumors are benign and 25 % are malignant.
• Primary cardiac sarcoma is a rare malignant cardiac neoplasm with a poor prognosis.
• No evidence-based guidelines exist regarding optimal surgical treatment of primary cardiac sarcomas.
• Systemic chemotherapy is indicated for patients with widespread or unresectable malignancies.

## 1 Introduction

Primary cardiac tumors are extremely rare, with a reported incidence of 0.001−0.03 % at autopsy. About 25 % of these tumors are malignant, 34 % are angiosarcomas, and 24 % are undifferentiated sarcomas [1]. Primary cardiac tumors cause nonspecific symptoms that delay their diagnosis, ultimately leading to more advanced disease. No evidence-based guidelines for surgical management of these tumors exist. At present, complete extensive surgical resection has been reported to increase postoperative survival [2,3]. However, the current survival rate remains poor [4,5]. Postoperative therapy prolongs survival after surgical resection of cardiac sarcoma; however, 5-year survival rates do not significantly differ from those observed with surgery alone [6]. Herein we report the outcome of a patient who received postoperative carbon-ion radiotherapy (CIRT) and chemotherapy for spindle cell sarcoma of the heart.

## 2 Case presentation

A 16-year-old female diagnosed with a left atrial (LA) tumor, suggestive of myxoma, was referred to our hospital. Electrocardiography conducted at high school enrollment revealed premature ventricular contraction. She was diagnosed with a LA tumor by transthoracic echocardiography (TTE) and was referred to us from a peripheral hospital. TTE showed a 16 × 20 mm mass arising from the anterior wall of the left atrium extending to the anterior mitral annulus. We diagnosed the patient with myxoma. Further assessment by enhanced computed tomography (CT) showed a large low-density mass (25 mm) in the left atrium. She underwent surgery 2 months later. TTE showed that the mass had increased to 19 × 28 mm.

**Fig. 1:**
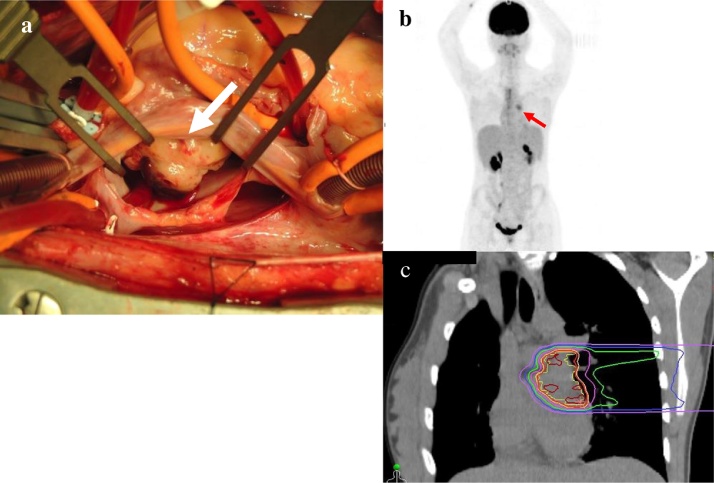
Perioperative imaging findings. (a) Surgical view of spindle cell sarcoma in left atrium (Tumor shown as white arrows). (b) PET showed residual disease in the heart but no distant metastasis (Residual tumor shown as red arrows). (c) Carbon-ion radiotherapy.

Tumor resection was performed. During surgery, the tumor was found to have an irregular LA surface of 30 × 30 mm, which invaded the left inferior pulmonary vein from the atrial septum (Fig. 1a). We suspected a malignant tumor and requested intraoperative evaluation. The diagnosis suggested sarcoma, resulting in incomplete resection. The histopathological diagnosis was high-grade spindle cell sarcoma. Postoperative 18-fluorodeoxyglucose (FDG) positron emission tomography (PET) initially showed localized FDG hyperaccumulation near the left pulmonary vein junction in the left atrium but no indication of distant metastasis. The patient then underwent CIRT, at a total dose of 64 Gy/16 fractions over 4 weeks, for residual lesions in the heart. PET following CIRT did not indicate any previous accumulation in the residual tumor. These findings suggested that tumor cell activity had stopped.

**Fig. 2:**
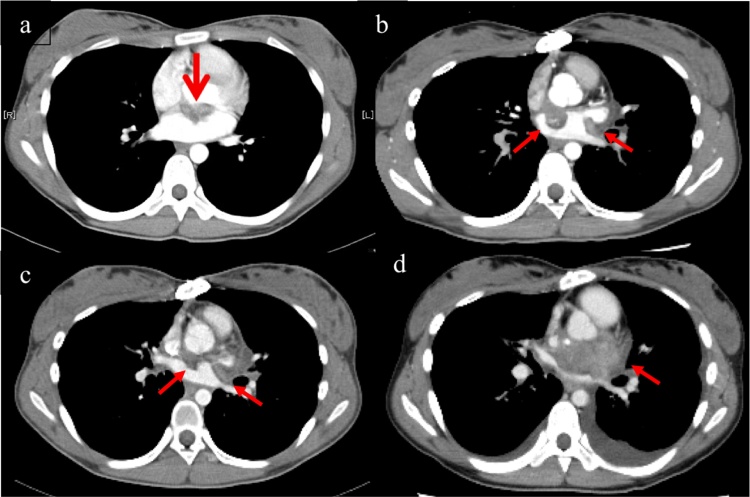
CT images. The tumor gradually grew (Tumor shown as red arrows). (a) Preoperative. (b) 1 year after surgery. (c) 2 year after surgery. (d) 4.5 year after surgery.

At 1 year after surgery, PET showed FDG accumulation in the left atrium and right clavicle outside the irradiation field, and she was diagnosed with recurrence and metastasis. CT showed tumor expansion (Fig. 2a). The mass in the left atrium was treated with CIRT (64 Gy/16 fractions over 4 weeks). She underwent right clavicle resection, and the histopathological results indicated sarcoma. The tumor disappeared after the second round of CIRT.

At 2 years after surgery, PET and CT showed that the LA tumor within the irradiation field had grown in size, with increased FDG accumulation compared with the previous examination (Fig. 2b). Because surgery was too risky, chemotherapy was initiated, but it was ineffective.

At 4 years, 4 months after surgery, she developed palpitations and dyspnea, and the tumor had spread rapidly. Two months later, the tumor had grown to 55 × 33 mm (Fig. 2c), although the mitral stenosis (mean pressure gradient: 10 mmHg) remained the same (Fig. 1b). She was hospitalized for heart failure and received medical treatment, but died at 4 years and 7 months after initial surgery.

## 3 Discussion

No established treatments for primary cardiac sarcomas are currently available. Therefore, systemic chemotherapy is indicated for patients with widespread or unresectable malignancies, and combined chemotherapy and radiation therapy is indicated for patients with primary cardiac lymphoma [1].

Simpson et al. reported that this disease significantly differs from other malignant tumors and requires amelioration of circulatory dysfunction caused by the tumor before any type of tumor control (both local and remote). These investigators recommend tumor resection as first-line treatment. They observed that the median survival of patients with primary cardiac tumors was 17 months versus 6 months after complete versus incomplete resection, respectively, suggesting that the extent of surgical resection contributes significantly to survival. However, 73 % of patients experienced recurrence despite complete resection, and 83 % of patients with incomplete resection experienced recurrence. Furthermore, death has been reported to occur due to obstruction of intracardiac blood flow because of local recurrence prior to observation of metastases [3].

Ramlawi et al. reported no significant differences in outcomes of cardiac sarcoma by side. Left-sided cardiac sarcomas are localized and less invasive, but may be associated with heart failure at an early stage. For cardiac sarcomas on the right side, survival rates were lower than those on the left side, because tumors were more likely to be extravasated, highly invasive, and metastatic [[7], [8], [9]]. The authors also reported a mean survival of 22 months in patients with sarcoma after cardiac autotransplantation and an operative mortality rate of 50 % in those who underwent cardiac autotransplantation combined with pneumonectomy. Furthermore, they recommended that patients should not be considered for surgery if the tumor invades one or more pulmonary veins and cannot be completely resected without pneumonectomy [10].

The role of adjuvant therapy following surgery in this setting has not yet been defined. Hendriksen et al. demonstrated that median survival was more than 2-fold longer in patients after postoperative therapy compared with resection alone (19 vs. 8 months; p = 0.026). In addition, multivariate analysis showed a significant association between an improved survival rate and postoperative therapy (p = 0.009), although no significant difference in 5-year overall survival rates was observed [6].

Few reports about the use of CIRT to treat cardiac sarcomas have been published. In the present case, complete resection with an appropriate resection margin was not possible, so postoperative CIRT treatment was performed. Our patient developed LA metastases 1 year after CIRT. However, she was treated again with CIRT and chemotherapy and survived 4 years and 7 months. It is unclear whether CIRT would be effective for other patients with high-grade spindle cell sarcoma, because we have treated only one case. However, the patient experienced relatively long survival compared with previously reported survival rates for incomplete resection. Thus, CIRT may be an effective treatment for primary cardiovascular sarcoma.

## 4 Conclusion

No established cures for spindle cell sarcoma are currently available. Relatively good results have been obtained with surgery and postoperative treatment. In the present case, incomplete surgical resection and postoperative carbon-ion radiotherapy were performed, resulting in relatively long survival.

## Declaration of Competing Interest

There are no conflicts of interest to report.

## Funding

None.

## Ethical approval

On the basis of this being a case report, the Institutional Review Board of the Dokkyo University does not mandate that ethical approval is required. Thus, this case report is exempt from the Institutional Review Board Approval process.

## Consent

Written informed consent was obtained from the patient for publication of this case report and accompanying images. A copy of the written consent is available for review by the Editor-in-Chief of this journal on request.

## Author’s contribution

Study concept, design, final proofreading: Ikuko Shibasaki.

Data collection: Shigeru Toyoda, Yusuke Takei.

Advised and designed the report: Masayuki Chida, Hirotsugu Fukuda.

## Registration of research studies

Not Applicable.

## Guarantor

Ikuko Shibasaki.

## Provenance and peer review

Not commissioned, externally peer-reviewed.

## References

[1] Butany J., Nair V., Naseemuddin A. (2005). Cardiac tumours: diagnosis and management. Lancet Oncol..

[2] Shanmugam G. (2006). Primary cardiac sarcoma. Eur. J. Cardiothorac. Surg..

[3] Simpson L., Kumar S.K., Okuno S.H. (2008). Malignant primary cardiac tumors: review of a single institution experience. Cancer.

[4] Okita Y., Miki S., Ueda Y. (1994). Recurrent malignant fibrous histiocytoma of the left atrium with extracardiac extension. Am. Heart J..

[5] Putnam J.B., Sweeney M.S., Colon R. (1991). Primary cardiac sarcomas. Ann. Thorac. Surg..

[6] Hendriksen B.S., Stahl K.A., Hollenbeak C.S. (2019). Postoperative chemotherapy and radiation improve survival following cardiac sarcoma resection. J. Thorac. Cardiovasc. Surg..

[7] Ramlawi B., Leja M.J., Abu Saleh W.K. (2016). Surgical treatment of primary cardiac sarcomas: review of a single-institution experience. Ann. Thorac. Surg..

[8] Blackmon S.H., Patel A., Reardon M.J. (2008). Management of primary cardiac sarcomas. Expert Rev. Cardiovasc. Ther..

[9] Kim M.P., Correa A.M., Blackmon S. (2011). Outcomes after right–side heart sarcoma resection. Ann. Thorac. Surg..

[10] Blackmon S.H., Patel A.R., Bruckner B.A. (2008). Cardiac autotransplantation for malignant or complex primary left-heart tumors. Tex. Heart Inst. J..

